# CDX2 increases SLC7A7 expression and proliferation of pig intestinal epithelial cells

**DOI:** 10.18632/oncotarget.8894

**Published:** 2016-04-21

**Authors:** Xiang-guang Li, Gao-feng Xu, Zhen-ya Zhai, Chun-qi Gao, Hui-chao Yan, Qian-yun Xi, Wu-tai Guan, Song-bo Wang, Xiu-qi Wang

**Affiliations:** ^1^ College of Animal Science, South China Agricultural University/National Engineering Research Center for Breeding Swine Industry, Guangzhou 510642, China

**Keywords:** CDX2, cell proliferation, intestinal epithelial cells, solute carrier family 7 member 7, pig

## Abstract

Nutrient absorption mediated by nutrient transporters expressed in the intestinal epithelium supplies substrates to support intestinal processes, including epithelial cell proliferation. We evaluated the role of Caudal type homeobox 2 (CDX2), an intestine-specific transcription factor, in the proliferation of pig intestinal epithelial cells (IPEC-1) and searched for novel intestinal nutrient transporter genes activated by CDX2. Our cloned pig *CDX2* cDNA contains a “homeobox” DNA binding motif, suggesting it is a transcriptional activator. CDX2 overexpression in IPEC-1 cells increased cell proliferation, the percentage of cells in S/G2 phase, and the abundance of transcripts of the cell cycle-related genes *Cyclin A2*; *Cyclin B*; *Cyclin D2*; *proliferating cell nuclear antigen*; and *cell cycle cyclin-dependent kinases 1, 2* and *4*, as well as the predicted CDX2 target genes *SLC1A1, SLC5A1* and *SLC7A7*. In addition, luciferase reporter and chromatin immunoprecipitation assays revealed that CDX2 binds directly to the *SLC7A7* promoter. This is the first report of CDX2 function in pig intestinal epithelial cells and identifies *SLC7A7* as a novel CDX2 target gene. Our findings show that nutrient transporters are activated during CDX2-induced proliferation of normal intestinal epithelial cells.

## INTRODUCTION

*CDX2* is a caudal-related homeobox gene coding for a homeodomain transcription factor [1]. Intestine-specific CDX2 is a critical regulator of gut development and homeostasis [2], containing a conserved homeotic DNA-binding site [3]. Simmini *et al*. [4] showed that inactivation of *CDX2* in intestinal stem cells re-specifies their identity and fate towards gastric stem cells. *CDX2* conditional knock-out mice fail to form mature endoderm in the intestinal epithelium, and intestinal stem cells lacking *CDX2* cannot differentiate into normal intestinal lineages in cultured crypts [5].

CDX2 is crucial to cell processes of the intestinal epithelium, including nutrient absorption, proliferation, adhesion, migration, apoptosis, and tumorigenesis, which are induced by transcriptional activation of relevant target genes [1, 2]. A study by Hinoi *et al*. [6] indicated CDX2 may control intestinal cell fate determination by activating transcription of liver intestine-cadherin. Moreover, CDX2 directly activates the cell adhesion genes Claudin-1 [7] and Claudin-2 [8]. CDX2 appears to stimulate intestinal epithelial cell differentiation by activating transcription of intestine-specific genes, such as sucrase-isomaltase and lactase phlorizin hydrolase, both of which are markers of the intestinal absorptive cells, and Mucin 2, a goblet cell marker involved in the formation of the mucosal barrier [2, 9].

CDX2 is a biomarker for gastrointestinal cancer [10], but little research has focused on its role in normal cells. Pigs are true omnivores and have intestinal physiologic processes, enteric microbiota composition, and disease pathogenicity similar to humans, such that there is a natural correlation between pig and human intestines [11]. No data are available concerning the role of CDX2 in pigs, and the complete coding sequence (CDS) sequence of pig *CDX2* was not yet obtained. The objective of the current study was to clone pig *CDX2*, evaluate its roles in pig intestinal epithelial cell proliferation, and look for novel intestinal nutrient transporter genes targeted by CDX2.

## RESULTS

### Cloning of pig CDX2

**Figure 1:**
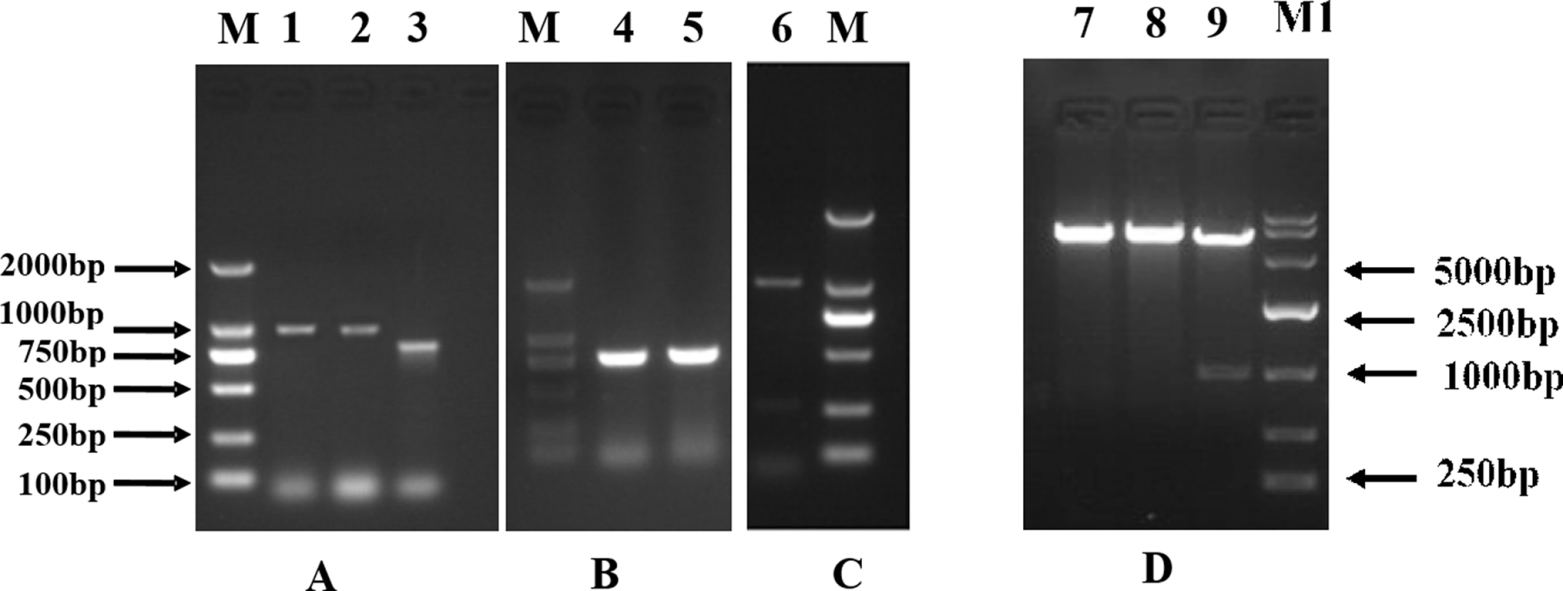
The cloning of pig CDX2 (A, B) and the identification of the recombinant plasmid CDX2-pcDNA3.1 (C, D) M: DNA Marker 2000; M1: DNA Marker 10000; Lane 1, 2: partial sequence 1 amplified by nest gene-specific primers; Lane 3: partial sequence 2 from conserved sequence; Lane 4, 5: 3′ RACE; Lane 6: PCR identification of the recombinant plasmid CDX2-pcDNA3.1; Lane 7, 8, 9: Enzyme digesting identification using *Kpn* I and *Xho* I of the recombinant plasmid CDX2-pcDNA3.1.

**Figure 2:**
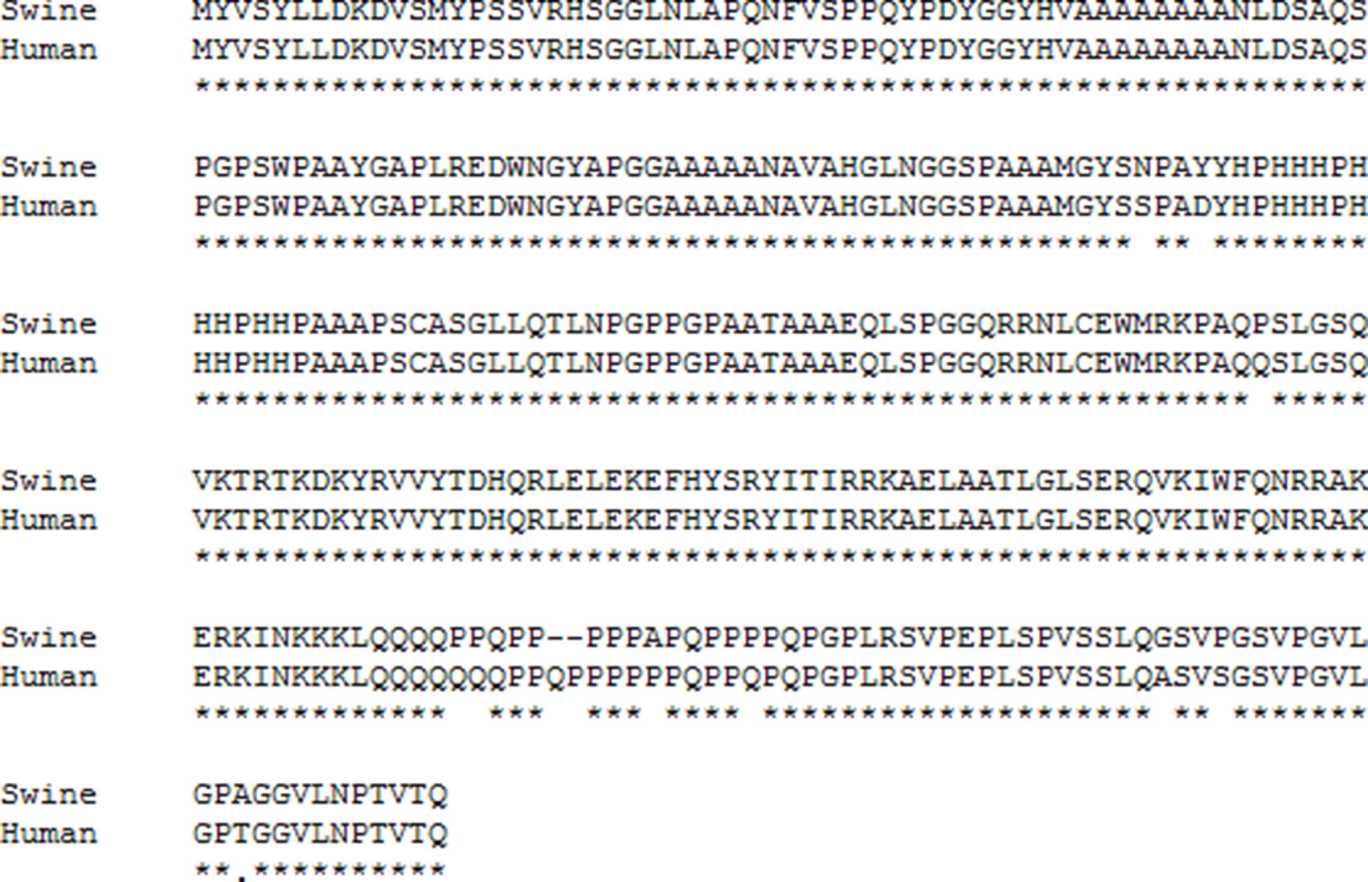
Comparison of the human and pig CDX2 protein sequences The pig CDX2 protein was predicted from the cloned nucleotide sequence using DNASTAR (www.dnastar.com), and the comparison was conducted using the same software. *indicates the same amino acid residue.

Based on the conserved sequences of human and mouse *CDX2*, two overlapping fragments of 972 bp and 786 bp were cloned (Figure 1A), yielding an 803 bp 3′-end sequence (Figure 1B). By aligning and splicing these sequences, we produced a 1934 bp pig *CDX2* cDNA (GenBank accession no. GU017420) with a complete open reading frame (ORF) containing a 974 bp 3′ UTR and a 981 bp ORF. The homology of the pig *CDX2* CDS with the human sequence was found to be 91.5%, while the protein homology was 96.17% (Figure 2). Bioinformatics performed using DNASTAR (www.dnastar.com) showed that pig CDX2 possesses a 61 amino acid “homeobox” DNA binding motif with a helix-turn-helix secondary structure, suggesting it is a transcriptional regulator of “downstream” genes. The *CDX2* overexpression vector was verified by RT-PCR with an M13 primer (Figure 1C) and identified through enzyme digest (Figure 1D).

### CDX2 overexpression increases the proliferation of IPEC-1 cells

**Figure 3:**
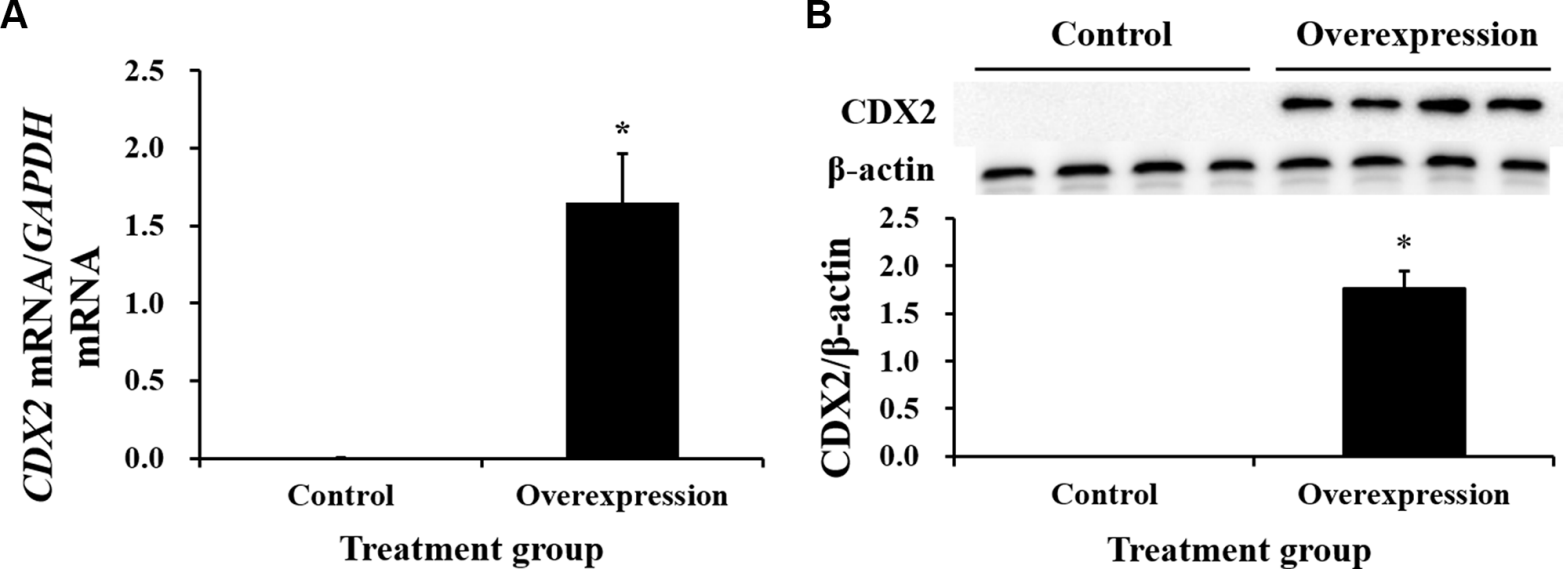
The mRNA abundance and protein expression of CDX2 in IPEC-1 are significantly increased by CDX2 overexpression Identification of the CDX2 mRNA abundance (**A**) *n* = 6) and protein level (**B**) *n* = 4) in the control and overexpression groups. The results were confirmed by three independent experiments with 6 (mRNA abundance) or 4 (protein level) samples per treatment. Representative results of the three independent experiments are shown. The bars are the means ± SE, *indicates a significant difference (*P* < 0.05).

**Figure 4:**
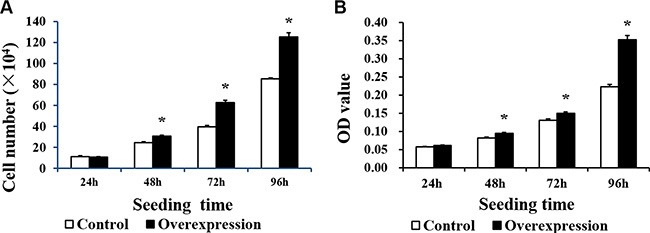
The proliferation of IPEC-1 is increased by CDX2 overexpression (**A**) The cell number of the overexpression group was higher than that of the control group at 48, 72 and 96 h after seeding, as assessed by the cell count assay (*n* = 6); (**B**) The OD value of the overexpression group was higher than the control at 48, 72 and 96 h after seeding, as assessed by the MTT assay (*n* = 20). The results were confirmed by three independent experiments with 6 (cell number) or 20 (the OD value) samples per treatment. Representative results of the three independent experiments are shown. The bars are the means ± SE, *indicates a significant difference (*P* < 0.05).

Neither CDX2 mRNA nor protein were detected in control cells (Figure 3). Both were highly increased in CDX2-pcDNA3.1-transfected cells. Cell count and MTT assays on pig intestinal epithelial cell line (IPEC-1) showed that CDX2 overexpression increased cell numbers (Figure 4A) and OD values (Figure 4B) (*P* < 0.05).

### CDX2 overexpression regulates cell cycle distribution of IPEC-1 cells

**Figure 5:**
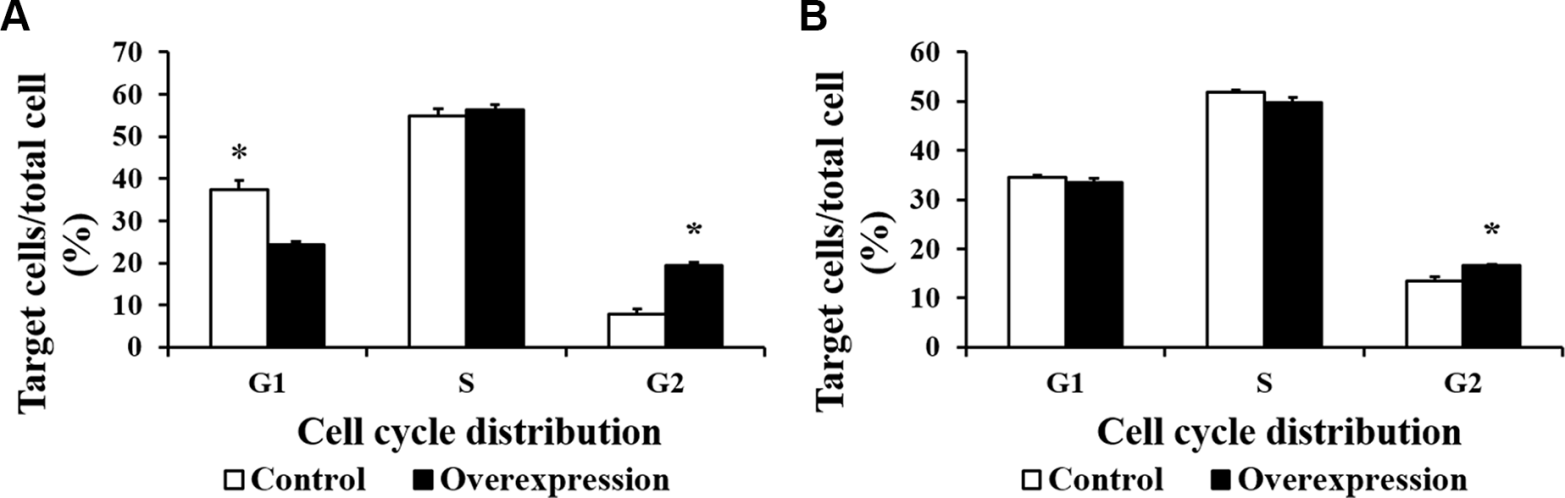
The cell cycle distribution of IPEC-1 is altered by CDX2 overexpression At 48 or 72 h after seeding, control and overexpression cells (10^4^ cells per sample) were collected to determine the cell cycle distribution using flow cytometry. (**A**) and (**B**) show the cell cycle distribution at 48 and 72 h after seeding, respectively. The results were confirmed by three independent experiments with 5 samples per treatment. The results shown are representative of three independent experiments. The bars are the means ± SE, *indicates a significant difference (*P* < 0.05).

To further investigate the proliferation differences induced by CDX2 overexpression, cell cycle analysis was conducted via flow cytometry (Figure 5). 48 h after seeding, the percentage of CDX2 overexpressing cells in G1 phase was lower (*P* < 0.05) and the percentage in G2 phase was higher (*P* < 0.05) relative to the control group. 72 h after seeding, the percentage of CDX2 overexpressing cells in G2 phase was higher (*P* < 0.05) than in the control group. Moreover, the percentage of cells in the S/G2 phases was higher (*P* < 0.05) in the overexpression group than in the control group at 48 and 72 h after seeding.

### Increased mRNA abundance of cell cycle related genes and CDX2 target genes

**Figure 6:**
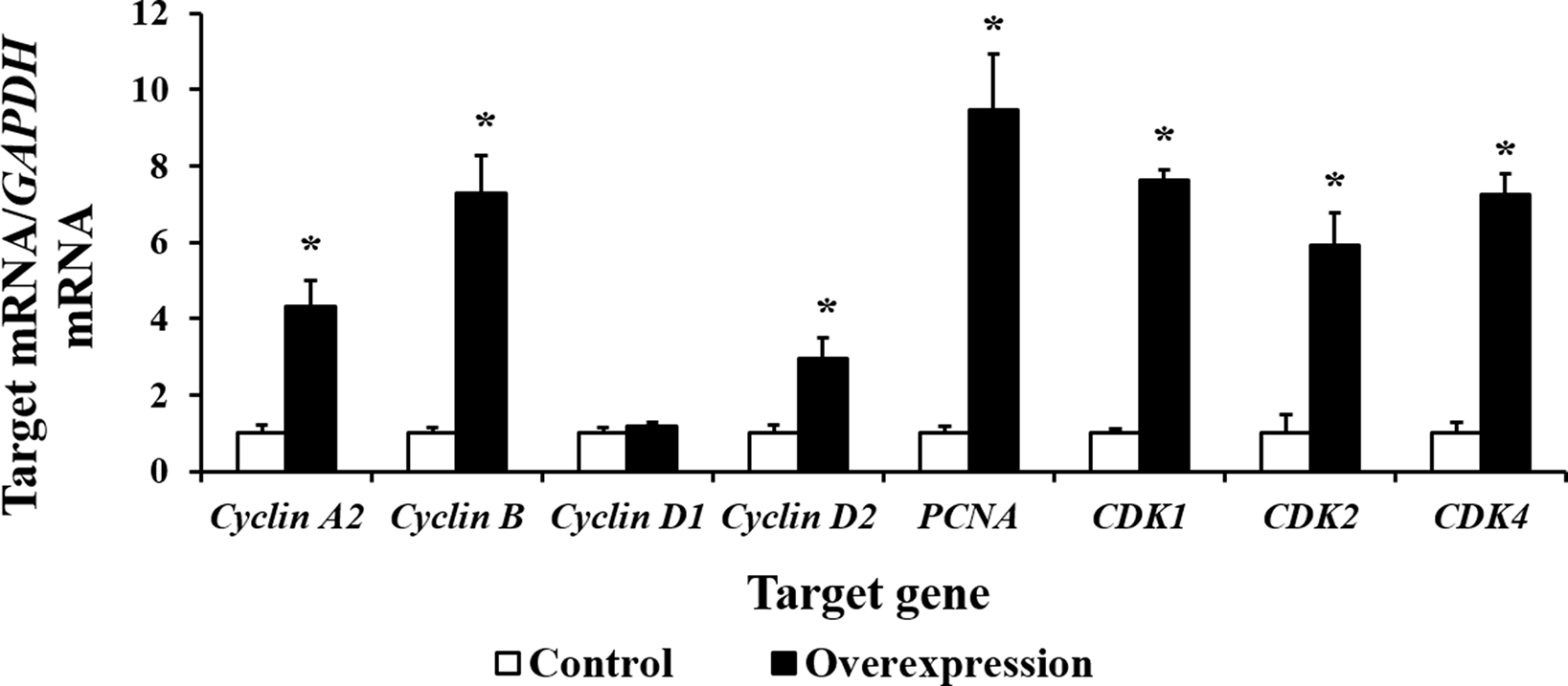
The mRNA abundance (*n* = 6) of cell cycle-related genes is increased by CDX2 overexpression At 72 h after seeding, control and overexpression cells were collected to determine the mRNA abundance of the genes of interest using a real-time polymerase chain reaction. *PCNA* = proliferating cell nuclear antigen; *CDK* = cell cycle cyclin-dependent kinase. The results were confirmed by three independent experiments with 6 samples per treatment. Representative results of three independent experiments are shown. The bars are the means ± SE, *indicates a significant difference (*P* < 0.05).

**Figure 7:**
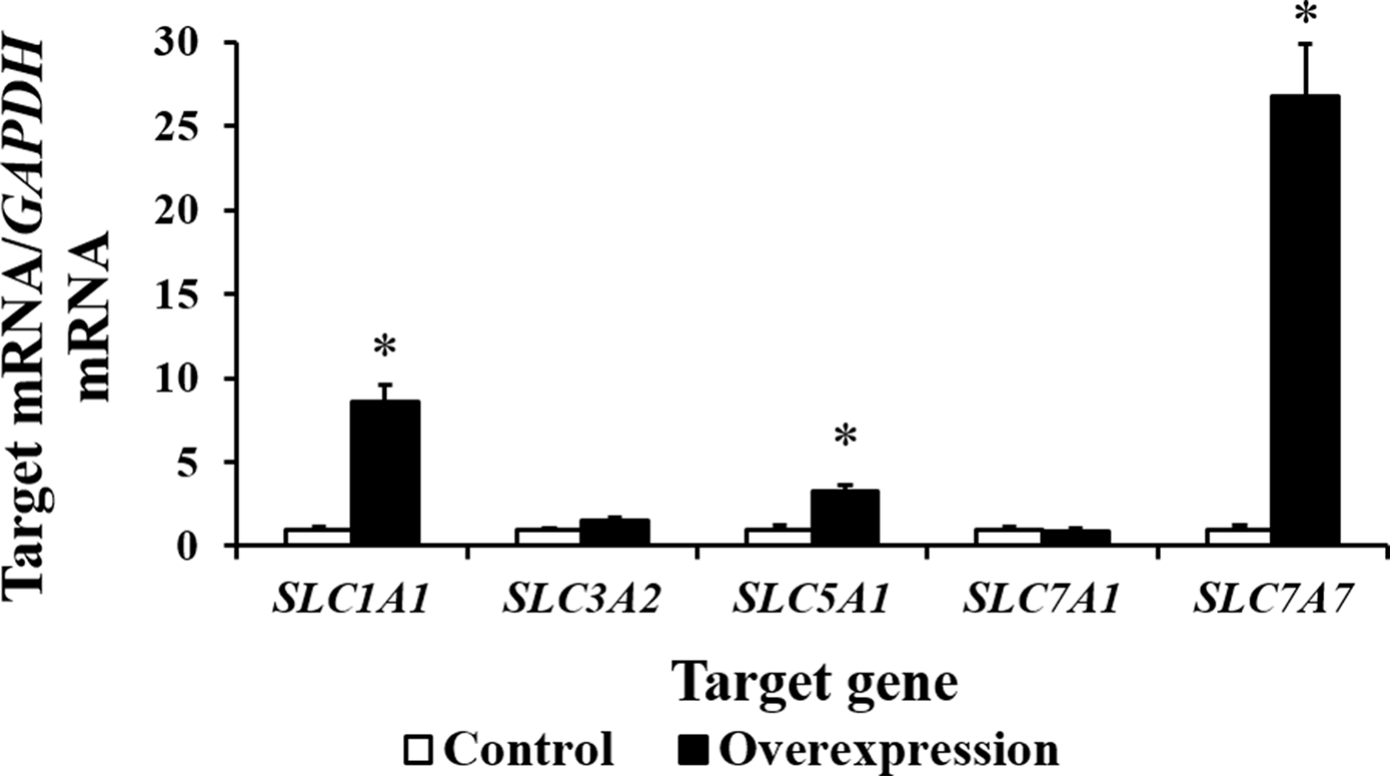
The mRNA abundance (*n* = 6) of several candidate target genes of CDX2 is increased by CDX2 overexpression At 72 h after seeding, control and overexpression cells were collected to determine the mRNA abundance of the nutrient transporter genes. *SLC1A1* = solute carrier (SLC) family 1 member 1; *SLC3A2* = SLC family 3 member 2; *SLC5A1* = SLC family 5 member 1; *SLC7A1* = SLC family 7 member 1; *SLC7A7* = SLC family 7 member 7. The results were confirmed by three independent experiments with 6 samples per treatment. Representative results of three independent experiments are shown. Bars represent the means ± SE, *indicates a significant difference (*P* < 0.05).

Our results show that CDX2 overexpression increased the mRNA levels of *Cyclin A2*, *Cyclin B*, *Cyclin D2*, proliferating cell nuclear antigen (*PCNA*), cell cycle cyclin-dependent kinase 1 (*CDK1*), *CDK2*, and *CDK4* (*P* < 0.05) (Figure 6). The results of the bioinformatics analysis using the WWW Promoter Scan, MatInspector, and ALGGEN-PROMO indicate that the intestinal nutrient transporter genes *SLC1A1*, *SLC3A2*, *SLC5A1*, *SLC7A1*, and *SLC7A7* are candidate target genes of CDX2. Real-time PCR showed that the mRNA abundance of *SLC1A1*, *SLC5A1*, and *SLC7A7* was higher in the overexpression group (*P* < 0.05) than in the controls (Figure 7). *SLC7A7* mRNA was increased approximately 27-fold, the largest change among the candidate target genes.

### CDX2 overexpression increases the activity of the *SLC7A7* promoter

**Figure 8:**
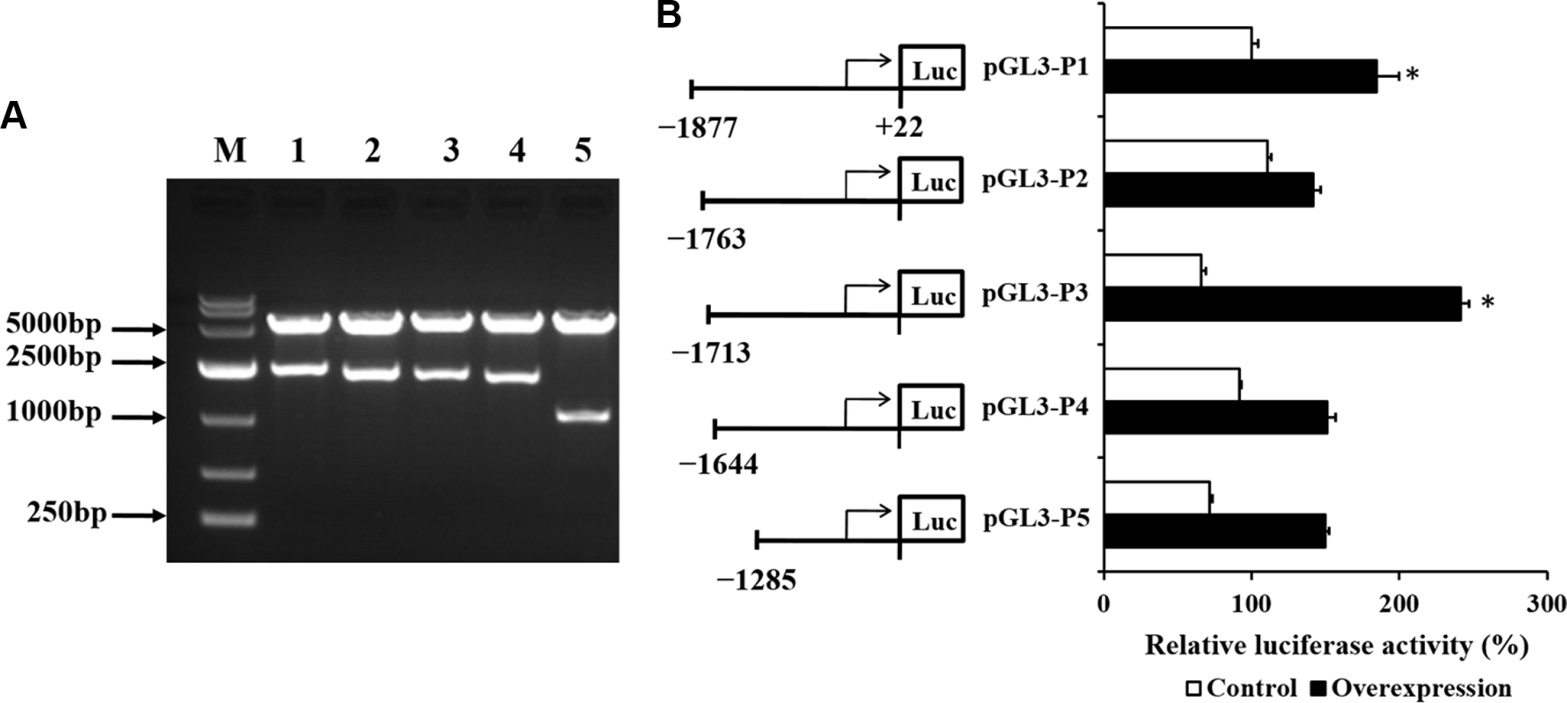
The promoter activity of the SLC7A7 deletion constructs in IPEC-1 cells transfected with empty vector or CDX2 recombinant vector are demonstrated (**A**) Identification of the *SLC7A7* promoters. (**B**) The promoter activity of the *SLC7A7* deletion constructs. The relative activity of each construct was normalized with the pRL-TK expression level. M = marker; 1-5 = pGL3-P1- pGL3-P5. Values are represented as mean ± SEM from at least three independent experiments.

To investigate whether CDX2 activates the *SLC7A7* promoter, the −1899/+22 luciferase reporter construct was transiently transfected into the control and overexpression cells. The primers and identification of the five reporter constructs are shown in Table 3 and Figure 8A, respectively. Figure 8B shows the luciferase reporter activities of the different promoter deletion constructs in the control and overexpression cells. *CDX2* transfection increased transcription from the *SLC7A7* promoter. The deletion constructs pGL3-P2, pGL3-P4, and pGL3-P5 reduced luciferase activities, suggesting CDX2 binds to the cis-regulatory elements in the −1877/+22 and −1713/+22 regions of the *SLC7A7* promoter.

### CDX2 binds the *SLC7A7* promoter directly

**Figure 9:**
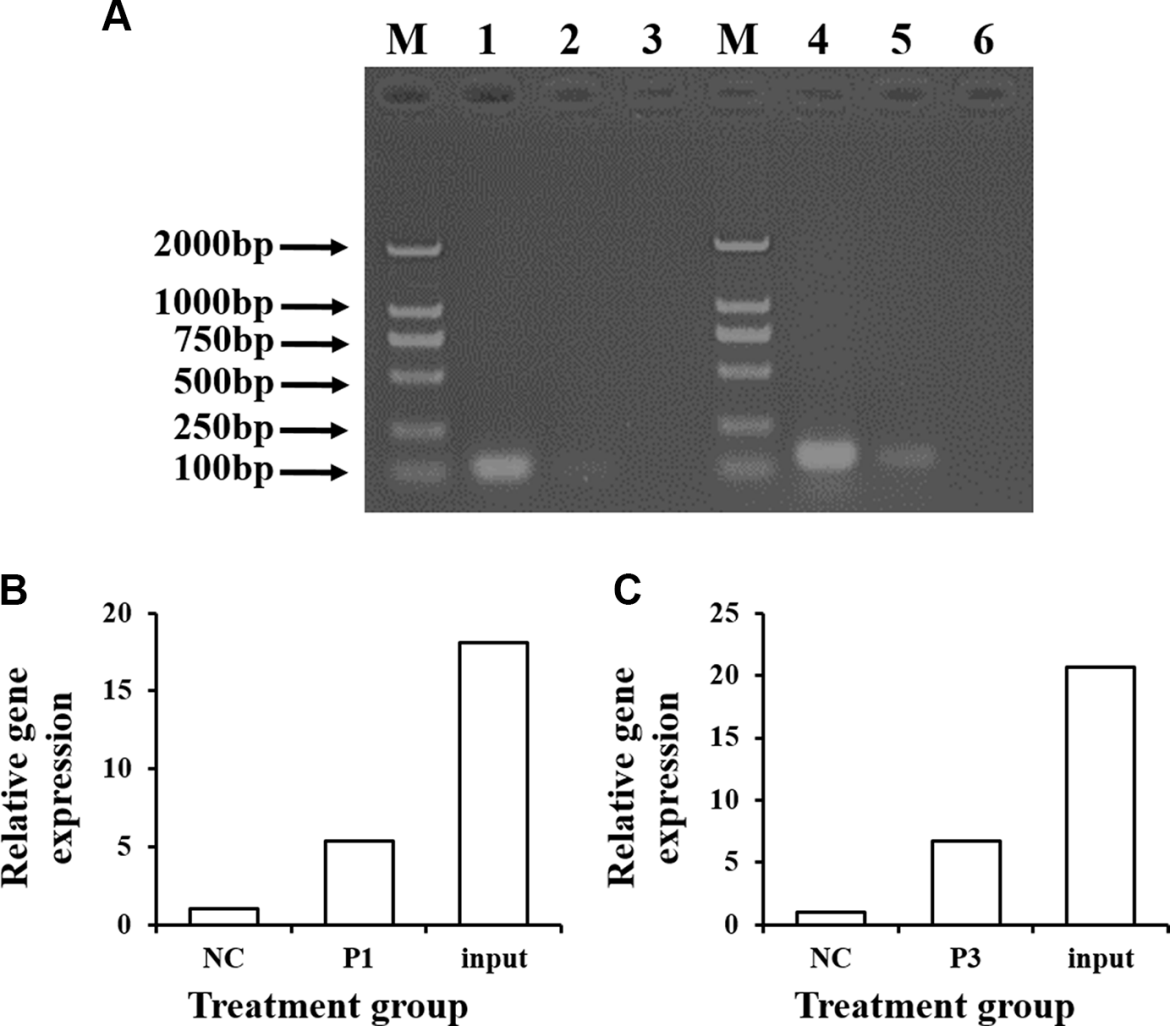
ChIP assays are performed to assess the specific binding of CDX2 to the pig *SLC7A7* promoter The cross-link between nuclear protein and genomic DNA from the overexpression IPEC-1 cells was immunoprecipitated with anti-CDX2 antibody and normal IgG (negative control, NC). Decross-link and real-time PCR-amplification with specific primers to detect the segments were performed. (**A**) PCR-amplification products were assessed by 1.5% agarose gel electrophoresis. M = marker; 1, 4 = Input, the control sample without immunoprecipitation; 2 = PCR-amplification product of the promoter segment 1 (−1877/+22, P1) in the SLC7A7 gene; 3, 6 = NC; 5 = PCR-amplification product of promoter segment 3 (−1713/+22) in the *SLC7A7* gene. (**B**) and (**C**) show the relative expression of the P1 and P3, respectively.

To test whether CDX2 activates the *SLC7A7* promoter through direct binding, we performed a ChIP-PCR analysis using a micrococcal nuclease digestion of the CDX2 overexpressing IPEC-1 cells. The promoter regions −1877/+22 and −1713/+22 of *SLC7A7* were enriched in anti-CDX2 but not in control immunoglobulin (IgG) immunoprecipitates (Figure 9), suggesting CDX2 binds the ChIP fragment region of the *SLC7A7* promoter.

## DISCUSSION

CDX2 regulates intestinal cell proliferation [12] by binding specific promoter sites and transcriptionally regulating target genes, including *SLC7A7*. In the present study, we cloned the complete ORF of pig *CDX2* and determined that it codes for a 61 amino acid “homeobox” DNA binding motif indicating that pig CDX2 is a transcriptional regulator, consistent its role in human and mouse intestines [13]. We proposed that it activates genes that increase normal pig intestinal epithelial cell proliferation.

The regulative roles of CDX2 in cell proliferation are variable. Collectively, CDX2 inhibits the proliferation of cancer cells, including HT-29 colon carcinoma cells [14], Caco2 cells [15], and BGC823 gastric cancer cells [16]. Its regulative role is controversial in IEC-6 cells. Suh *et al*. [17] reported that the conditional expression of CDX2 in IEC-6 cells inhibits cell proliferation; however, other study shows that the conditional expression of CDX2 in IEC-6 cells increases cell proliferation [18]. In the present study, CDX2 overexpression increased IPEC-1 cell proliferation and led to a greater percentage of cells in the G2 and S/G2 phases. Moreover, the mRNA abundance of *PCNA*; the cell cycle promoters *Cyclin A2*, *Cyclin B*, and *Cyclin D2*; and *CDK1*, *CDK2*, and *CDK4* were increased following CDX2 overexpression. These results collectively demonstrate that CDX2 promotes proliferation in pig intestinal epithelial cells.

Nutrient absorption, mediated by nutrient transporters, supports the growth of intestinal epithelial cells. Our previous studies have shown that the gene expression of *SLC1A1*, *SLC7A9*, and *SLC15A1* in chicks [19, 20] and *SLC7A9*, *SLC1A1, SLC43A2*, *SLC15A1*, *SLC9A2*, *SLC9A3*, and *SLC7A6* in pigeons positively correlates with the growth of embryonic intestines [21, 22]. In addition, the inhibition of *SLC1A1* decreased the proliferative activity of the IPEC-1 cells, as expected [23]. We used bioinformatics analysis tools to predict candidate CDX2 target genes from among the nutrient transporters located in the pig intestinal epithelium. CDX2 binding sites were identified in the promoters of pig *SLC1A1*, *SLC3A2*,*SLC5A1*, *SLC7A1*, and *SLC7A7*. The gene expression levels of *SLC1A1*, *SLC5A1*, and *SLC7A7* were increased by CDX2 overexpression; *SLC7A7* was increased approximately 27-fold. SLC7A7 is a member of System y^+^L, which was originally identified as a transport system that transports both neutral and basic amino acids in the erythrocyte plasma membrane [24]. When SLC7A7 mediates a heteroexchange, the influx of substrate amino acids is accompanied by the efflux of basic amino acids [25]. Enhancement of SLC7A7 expression may contribute to the improvement of intestinal morphology and absorptive function [26, 27].

Using reporter assays, we found that CDX2 increased *SLC7A7* promoter activity in IPEC-1 cells. Deletion analysis revealed that the CDX2 responsive regions were located between bases −1877 to −1763 and −1713 to −1644 relative to the transcription start site. The results of a previous study showed that CDX2 overexpression in the colon cancer cell line SW480 improved the Claudin-1 gene promoter activity by 6-fold but only prompted a 4-fold increase in HCT116 cells [7]. CDX2 binds the promoter region of *SLC5A8* and increases its expression in cultured cells and in colonic epithelium. Thus, CDX2 may increase the uptake of SLC5A8 transporter substances in the colon [28]. In this study, after CDX2 was transfected into IPEC-1 cells, the *SLC7A7* gene promoter activity increased 2-fold. Like IEC-6 [17] and HIEC cells [29], wild-type IPEC-1 cells do not express CDX2. ChIP assays showed that CDX2 binds directly to *SLC7A7* at −1877/+22 and −1713/+22. Currently, the regulation of gut health and nutrient utilization in pigs is believed to depend mainly on exogenous additives, such as amino acids [30, 31], fatty acids [32], and probiotics [33]. The results of the current study provide additional information on the regulation of nutrient transporter genes in intestinal epithelial cells.

In conclusion, we are the first group to clone a pig *CDX2* cDNA containing its full-length ORF sequence. CDX2 overexpression increases the cell proliferative activity and gene expression of several nutrient transporters in the pig intestinal epithelium. Moreover, we report for the first time that CDX2 binds directly to the promoter region of *SLC7A7*, and increases gene transcription. According to currently available data, nutrient transporters, particularly *SLC7A7*, are involved in the CDX2-mediated improvement of cell proliferation. However, additional studies will be valuable in confirming this result.

## MATERIALS AND METHODS

### Intestinal tissue sample preparation

Three male and 3 female 5-d-old Landrace piglets were euthanized with sodium pentobarbital following the guidelines of the Animal Care Committee of South China Agricultural University (Guangzhou, China). The duodena, jejuna, and ilea were rapidly removed, washed with ice-cold phosphate buffered saline (PBS), and frozen in liquid nitrogen.

### RNA extraction and first-strand cDNA synthesis

Total RNA was isolated from the duodenum, jejunum, and ileum mixture of the piglets or IPEC-1 cells using TRIzol Reagent (Invitrogen, Carlsbad, CA, USA). The concentration and purity of the RNA sample was determined by measuring the optical density using a NanoDrop 2000 UV-Vis spectrophotometer (Thermo, Wilmington, MA, USA). The integrity of the RNA was assessed with 1.5% agarose gel electrophoresis. The cDNA was synthesized from 2 μg of DNA-free RNA using N10 primers (Sangon, Shanghai, China) and Moloney murine leukemia virus reverse transcriptase (Invitrogen, Carlsbad, CA, USA), according to the manufacturer's instructions. The synthesized cDNA was stored at −20°C for further processing.

### Cloning the full-length cDNA of pig CDX2

Primers were designed using Primer Premier 5.0 (Premier Biosoft, Palo Alto, CA, USA). Two pairs of nested gene-specific primers (*CDX2*-A, *CDX2*-A-in; Table 1) and one primer pair (*CDX2*-B) were first designed based on the human (NM_001265.4) and mouse (NM_023963.1) conserved sequences. After comparison with the human sequence, a contig was made of the two fragments. Then, the 3′ gene-specific primer (Table 1) was designed to extend the cDNA end with a SMART RACE cDNA Amplification Kit (Clontech, Palo Alto, CA, USA) based on the contig sequence. The amplification reaction for the GC-rich region of the full-length cDNA was performed with EX-Taq Polymerase (TaKaRa, Shiga, Japan) in the presence of GC-buffer 1 (TaKaRa, Shiga, Japan). After aligning, the 3′ sequence and the previous contig sequence were assembled to the full-length *CDX2* cDNA sequence. The open reading frame (ORF) was later cloned to verify the results.

**Table 1:** Primers used for cloning the CDS of *CDX2*

Gene	Primer sequence	Product size (bp)
GSP2	5′-GGGGAGCATGGACTCTGCTAGAATCC-3′	
NGSP2	5′-CTGGTCTGACCTTCTGCCAGTAAGGAG-3′	
*CDX2*-A	F: 5′-GGAAGGAGGTGGGAGGAAAGAAG-3′	1295
	R: 5′-GGGAAGTTGGTGAACATTTGTGGG-3′	
*CDX2*-A-in	F: 5′-GTTTGCCGTCGGGACCTCG-3′	972
	R: 5′-GCCAACTGTCCGTCACTGGGTG-3′	
*CDX2*-B	F: 5′-CGTGCTAAACCCCACCGTCAC-3′	786
	R: 5′-TGACAGGAAGTCCAGGTTGGC-3′	
*CDX2*-ORF	F: 5′- ATGTACGTGAGCTACCTCCTGG-3′	936
	R: 5′-TCACTGGGTGACGGTGGG-3′	
*CDX2* CDS-Flag	F:5′-CGCGGATCC**GATTACAAGGATGACGACGATAAGTAA**AT GTACGTGAGCTACCTCCTGG-3′	981
	R:5′-CCGGAATTCTCACTGGGTGACGGTGGG-3′	

GSP2 = 3′ RACE out primer; NGSP2 = 3′ RACE nested primer; *CDX2*-A = out primers for cloning *CDX2* partial 1;*CDX2*-A-in = nested primers for cloning *CDX2* partial 1; *CDX2*-B = primers for cloning *CDX2* partial 2; *CDX2*-ORF = primers for cloning *CDX2* ORF; *CDX2* CDS-Flag = primers for cloning *CDX2* CDS containing flag (boldface sequence); the underlined sequences in the F and R primers are the enzyme digest sites of BamHI and EcoRI, respectively; the sequences in bold are Flag-tag sequences.

### Construction of the CDX2-pcDNA3.1 plasmids

The CDS of *CDX2* was amplified with primers (Table 1), which contained *Eco*RI and *Bam*HI enzyme digest sites. The purified PCR product was cloned into the *Eco*RI/*Bam*HI site of the pcDNA3.1+ vector. The resulting plasmid CDX2-pcDNA3.1 was transferred into DH5α and cultured in a LA medium plate for 14–16 h at 37°C. Several colonies were chosen to expand the culture and extract their plasmids for PCR, enzyme digestion, and subsequent sequencing identification. The purification of the PCR product and plasmid was performed using the TIANgel Midi Purification Kit (Tiangen, Beijing, China) and EndoFree Maxi Plasmid Kit (Tiangen, Beijing, China), respectively, according to the manufacturer's protocol.

### Cell culture and the construction of stable cell strains

IPEC-1 cells were obtained from Texas A&M University [34] and maintained in growth medium [Dulbecco's modified Eagle's medium (DMEM) supplemented with 5% fetal bovine serum (FBS), epidermal growth factor (EGF, 5 μg/L), insulin (5 μg/mL), transferrin (5 μg/mL), selenium (5 ng/mL), penicillin (50 μg/mL) and streptomycin (4 μg/mL)] at 37°C in a 5% CO2 incubator (Shellab, Cornelius, OR, USA). DMEM, FBS, EGF, and penicillin/streptomycin were obtained from Gibco (Carlsbad, CA, USA). Insulin, transferrin, and selenium were purchased from ScienCell (Carlsbad, CA, USA). IPEC-1 cells were seeded onto 6-well plates (#3516, Corning, NY, USA) at a density of 1 × 10^6^ cells/well and transfected with the basic-pcDNA3.1 or CDX2-pcDNA3.1 plasmid using Lipofectamine 2000 (Invitrogen, CA, USA) on the following day, according to the manufacturer's recommendations. 6 h after transfection, the cells were detached with 0.25% trypsin (Sigma, St Louis, MO, USA) and reseeded onto 6-well plates at a ratio of 1:10. G418 was then added to the medium at a final concentration of 400 μg/mL. After 7 d, the G418 concentration was decreased to 200 μg/mL. At this point, the cells of non-transfected group had all died. Fourteen days later, the surviving clones were selected, and CDX2 mRNA and protein expression was analyzed to confirm the positive clones. In the following experiments, the IPEC-1 cells transfected with basic-pcDNA3.1 and CDX2-pcDNA3.1 were named the control group and the overexpression group, respectively.

### Cell proliferation analysis

To assess cell proliferation, cell count and MTT assays were completed as previously described [35, 36].

### Cell count assay

Control and overexpression cells were cultured in 6-well plates (#3516, Corning, NY, USA) at a density of 6 × 104 cells/mL in growth medium and counted using trypan blue. Briefly, cells were detached with 0.25% trypsin (Sigma, St Louis, MO, USA) for 3–5 min at 37°C after washing twice with PBS and then blocked with an equal volume of growth medium. The number of viable cells was determined using a hemocytometer under an Inversion Microscope System (Nikon, Japan) at 24, 48, 72, and 96 h after seeding. The results were confirmed by three independent experiments with three samples per treatment. Representative results of the three independent experiments are shown.

### The MTT assay

Control and overexpression cells were cultured in 96-well plates (#3599, Corning, NY, USA) at a density of 5×10^3^ cells/ mL in growth medium. 20 μl of 3-(4,5-dimethylthiazol-2-yl)-2,5-diphenyl tetrazolium bromide (MTT) (5 mg/mL; Sigma, St Louis, MO, USA) solution was added to each well and incubated for 4 h. The plates were centrifuged at 3500 ×g for 20 min at room temperature before the untransformed MTT solution was carefully removed. Then, 150 mL of dimethylsulfoxide (DMSO) was added to each well. After a 10-min incubation at room temperature, the OD value was evaluated on a microplate reader at a wavelength of 490 nm. The results were confirmed by three independent experiments with 20 samples per treatment.

### Cell cycle distribution analysis

In order to determine the effects of CDX2 overexpression on the cell cycle distribution, the control and overexpression cells cultured in the 6-well plates were collected at 48 or 72 h after seeding. Flow cytometry was performed as previously described [37]. Briefly, the cells were washed twice with ice cold PBS and fixed with 70% ice-cold ethanol overnight at −20°C until further processing. The cells were centrifuged at 1500 × g for 5 min at 4°C and were subsequently re-suspended in 1 mL PBS. They were then treated with 100 μL of 200 mg/mL DNase-free RNaseA and incubated at 37°C for 30 min. Finally, the cells were treated with 100 μL of 1 mg/mL propidium iodide (PI) and incubated at room temperature (25°C) for 10 min. Then, 10^4^ cells per sample were subjected to flow cytometry using a Becton Dickinson FACScan (BD Biosciences, Franklin Lake, NJ, USA) at 500 mW. The results were confirmed by three independent experiments with 5 samples per treatment. Representative results of the three independent experiments are shown.

### Real-time polymerase chain reaction (real-time PCR)

The control and overexpression cells were cultured in 6-well plates (#3516, Corning, NY, USA) at a density of 6 × 10^4^ cells/mL in growth medium. At 72 h after seeding, the cells were collected for real-time PCR analysis, which was performed on a StratageneMxPro 3005P thermocycler (Agilent Technologies, Santa Clara, CA, USA) using the SYBR Green Real-Time PCR Master Mix (TOYOBO, Tokyo, Japan). The gene-specific primers used for real-time PCR are detailed Table 2. The results were confirmed by three independent experiments with three samples per treatment. Representative results of the three independent experiments are shown.

**Table 2:** Primers used for quantitative real-time PCR

Gene/ Accession	Primer sequence	Product size (bp)
*CDX2*	F: 5′-GTCGCTACATCACCATTCGG-3′	110
	R: 5′-GATTTTCCTCTCCTTCGCTCT-3′	
*GAPDH* (NM_001206359.1)	F: 5′-AGGTCGGAGTGAACGGA-3′	145
	R: 5′-TGGGTGGAATCATACTGG-3′	
*SLC1A1* (NM_001164649)	F: 5′-ATAGAAGTTGAAGACTGGGAAAT-3′	199
	R: 5′-GTGTTGCTGAACTGGAGGAG-3′	
*SLC3A2* (EU587016)	F: 5′-CTCGAACCCACCAAGGAC-3′	174
	R: 5′-GAGGTGAGACGGCACAGAG-3′	
*SLC5A1* (NM_001012297)	F: 5′- GCTGTTCATCCTGGTGCT-3′	381
	R: 5′- ACATCTGGAATGGGCTTG -3′	
*SLC7A1* (NM_001012613)	F: 5′-TGCCCATACTTCCCGTCC-3′	192
	R: 5′-GGTCCAGGTTACCGTCAGG-3′	
*SLC7A7* (EU390782)	F: 5′-CCATCACCTTTGCCAACTACG-3′	225
	R: 5′-AGGCTCCCTGACCAAGTCTAAC-3′	
*PCNA* (DQ473295.1)	F: 5′-TACGCTAAGGGCAGAAGATAATG-3′	192
	R: 5′-CTGAGATCTCGGCATATACGTG-3′	
*Cyclin A2* (NM_001177926.1)	F: 5′-TTAGGGAAATGGAGGTTA-3′	157
	R: 5′-TAGTTCACAGCCAAATGC-3′	
*Cyclin B* (GQ184631.1)	F: 5′-TGGCTAGTGCAGGTTCAG-3′	199
	R: 5′-CAGTCACAAAGGCAAAGT-3′	
*Cyclin D1* (AK400348.1)	F: 5′-GCGAGGAACAGAAGTGCG-3′	192
	R: 5′-TGGAGTTGTCGGTGTAGATGC-3′	
*Cyclin D2* (NM_214088.1)	F: 5′-TTACCTGGACCGCTTCTTG-3′	155
	R: 5′-GAGGCTTGATGGAGTTGTCG-3′	
*CDK1* (NM_001159304.2)	F: 5′-CCCTCCTGGTCAGTTCAT-3′	166
	R: 5′-TAGGCTTCCTGGTTTCC-3′	
*CDK2* (XM_003481615.1)	F: 5′-AAACAAGTTGACGGGAGA-3′	297
	R: 5′-GTGAGAATGGCAGAAAGC-3′	
*CDK4* (NM_001123097.1)	F: 5′-GCATCCCAATGTTGTCCG-3′	126
	R: 5′-GGGGTGCCTTGTCCAGATA-3′	
*SLC7A7-P1*	F: 5′-GAGGCTGTAGGGTAGCGATA-3′	132
	R: 5′-CCTACTCATTCTGAAATCCAACT-3′	
*SLC7A7-P3*	F: 5′-GAGTAGGGTTTATTTCTGTTGGT-3′	138
	R: 5′-CTAAGATTTCACTGAGTTATGCTG-3′	

*GAPDH* = glyceraldehyde-3-phosphate dehydrogenase; *SLC1A1* = solute carrier (SLC) family 1 member 1; *SLC3A2* = SLC family 3 member 2; *SLC5A*1 = SLC family 5 member 1; *SLC7A1* = SLC family 7 member 1; *SLC7A7* = SLC family 7 member 7; *PCNA* = proliferating cell nuclear antigen; *CDK* = cell cycle cyclin-dependent kinase; *SLC7A7*-P1 = *SLC7A7* promoter 1 (−1877/+22); *SLC7A7*-P3 = *SLC7A7* promoter 3 (−1713/+22).

### Western blot

The control and overexpression cells were cultured in 6-well plates (#3516, Corning, NY, USA) at a density of 6 × 10^4^ cells/mL in growth medium. At 72 h after seeding, the cells were collected for Western blot analysis. The cells were homogenized in a buffer containing 50 mmol/L tris-HCl (pH 7.5), 150 mmol/L NaCl, 1 mmol/L phenyl-methylsulfonyl fluoride (PMSF) and 3 mmol/L ethylene diamine tetraacetic acid (EDTA) at 4°C. The homogenates were then centrifuged at 12000 × g for 15 min to obtain the supernatant proteins. The protein concentration was determined using a BCA Protein Assay Reagent Kit (Pierce, Rockford, IL, USA). After boiling for 5 min, the samples (20 μg each) were subjected to 10% SDS-polyacrylamide gel electrophoresis (PAGE) at 80 V for 20 min and 110 V for 70 min using Tris-glycine running buffer. The separated proteins were transferred onto polyvinylidene difluoride (PVDF) membranes by electroblotting in a transfer buffer (25 mmol/L Tris base, 192 mmol/L glycine, and 10% methanol, pH 8.3). The membranes were blocked in 5% BSA in TBST buffer (20 mmol/L Tris, 500 mmol/L NaCl, 0.05% Tween-20, pH 7.6) for 3 hours at room temperature and then incubated overnight at 4°C with primary antibodies (anti-CDX2, Cell Signaling Technology, Beverly, MA, USA, 1:2000; anti-β-actin, Santa Cruz Biotechnology, Dallas, TX, USA, 1:2000). The next day, the membranes were washed 6 times for 5 min each with TBST buffer, followed by incubation with horseradish peroxidase-labeled anti-goat IgG (Bioworld Technology, Louis Park, MN, USA; 1:5000) for 1 h at room temperature. Immunoreactivity was detected using the ECL Plus chemiluminescence detection kit (Beyotime, Shanghai, China) in a FluorChem M system (Cell Biosciences, San Leandro, CA, USA). The density of the bands was analyzed using Image Analysis Software (Tanon, Shanghai, China). Unless otherwise indicated, all other chemicals were purchased from Sigma-Aldrich (St. Louis, MO, USA). The results were confirmed by three independent experiments with 6 samples per treatment. Representative results of the three independent experiments are shown.

### Bioinformatics

The WWW Promoter Scan (http://www-bimas.cit.nih.gov/molbio/proscan/) and MatInspector (http://www.genomatix.de) bioinformatics programs were used to complete the analysis of the promoter regions of the nutrient transporter genes in the pig intestine and to further predict the novel target genes of CDX2.

### Construction of reporter plasmids and cell transfection

The promoter region of pig *SLC7A7* was cloned and inserted into the *Kpn*I/*Mlu*I sites of pGl3-basic luciferase (Promega, Madison, WI, USA) to construct the pGL3-P1 plasmid. Subclones extending to positions −1763, −1713, −1644, and −1285 were constructed by deletion with restriction enzymes and re-ligation or by cloning the PCR sub-fragments into the pGl3-basic plasmid to produce pGL3-P2, pGL3-P3, pGL3-P4, and pGL3-P5 plasmids, respectively. (Table 3; Figure 8). Cells were seeded onto 24-well plates at a density of 10^5^ cells/mL for 48 h, after which the cells reached approximately 70–80% confluence. Cells were then transfected with 0.1 mg purified recombinant report plasmids and 0.1 mg internal control reporter pRL-TK vector in serum-free medium using the Lipofectamine 2000 reagent (Invitrogen, Carlsbad, CA, USA).

**Table 3:** Primers used for the construction of the truncated mutants of the SLC7A7 promoter region

Gene/ Accession	Primer sequence	Product size (bp)
pGL3-P1-F	F: 5′-**CGG**GGTACCGAGGCTGTAGGGTAGCGATAA-3′	1899
pGL3-P2-F	F: 5′**CGG**GGTACCGATTTCAGAATGAGTAGGGTTTAT-3′	1785
pGL3-P3-F	F: 5′-**CGG**GGTACCATTTAAGTACACTCTAACAACAG-3′	1735
pGL3-P4-F	F: 5′-**CGG**GGTACCGCCAGTTCAGCATAACTCAG-3′	1666
pGL3-P5-F	F: 5′-**CGG**GGTACCCAACCTTCCTGCCTTTCTT-3′	1307
pGL3-P-R	R: 5′-**CCG**CTCGAGCTCACAACACCACTCTTTTCC-3′	

pGL3-P1-F = forward primer of recombinant pGL3-P1 (−1877/+22 in *SLC7A7* promoter region); pGL3-P2-F = forward primer of recombinant pGL3-P2 (−1763/+22 in *SLC7A7* promoter region); pGL3-P3-F = forward primer of recombinant pGL3-P3 (−1713/+22 in *SLC7A7* promoter region); pGL3-P4-F = forward primer of recombinant pGL3-P4 (−1644/+22 in *SLC7A7* promoter region); pGL3-P5-F = forward primer of recombinant pGL3-P5 (−1285/+22 in *SLC7A7* promoter region); pGL3-P-R = reverse primer of pGL3-P1 to pGL3-P5; the underlined sequences in the F and R primers show the enzyme digest sites of *Bam*HI and *Eco*RI, respectively; the sequences in bold are the protective bases.

### Luciferase reporter assay

The activities of firefly and renilla luciferase in the cell lysates were measured using the Dual-Luciferase Reporter Assay System (Promega, Madison, WI, USA) according to the manufacturer's instructions. Cells were washed with PBS twice and then lysed with lysis buffer at room temperature for 15 min at 48 h after transfection. 20 μL of the cell lysate was transferred to a 96-well plate, and then 100 μL of Luciferase Assay Reagent II and 100 μL of Stop & Glo Reagent were sequentially added. The resulting intensity of the firefly and renilla luciferase was measured using a luminometer (BioTek, Winnoski, VT, USA). As a transfection efficiency control, the firefly luciferase activity was normalized to the renilla luciferase activity. All experiments were performed in duplicate and repeated at least three times with positive (pGL3-Control, Promega) and negative (pGL3-Basic, Promega) controls.

### Chromatin immunoprecipitation (ChIP) assay

Standard ChIP assays were performed using the Pierce^™^ Agarose ChIP Kit (#26156; Thermo Fisher Scientific, USA) according to the manufacturer's instructions. The Overexpression cells were seeded in a culture plate for 48 h to reach 50% confluence. The cross-linking between the nuclear protein and genomic DNA was performed in the growth medium with 1% formaldehyde supplementation at room temperature for 10 min. The cells were harvested and lysed with Lysis Buffer I. Chromatin was digested with MNase for 15 min at 37°C and incubated with CDX2 antibody or normal rabbit IgG overnight at 4°C. The antibody-chromatin complex beads were captured by Protein A/G agarose. DNA was separated from the beads and eluted using IP Elution Buffer for 40 min at 65°C. The DNA was further purified using a DNA Clean-up Column. Real-time PCR amplification was performed on a Stratagene MxPro 3005P thermocycler (Agilent Technologies, Santa Clara, CA, USA) using gene-specific primers for *SLC7A7*-P1 and *SLC7A7*-P3 (Table 2). PCR products were analyzed by electrophoresis on 1.5% agarose gels.

### Statistical analysis

Data were processed using the statistical software package SAS 9.1.3 (SAS Institute Inc., NC) and expressed as the mean ± SE. Variance analysis was completed using a GLM procedure. *P* < 0.05 was considered a significant difference between the groups.
